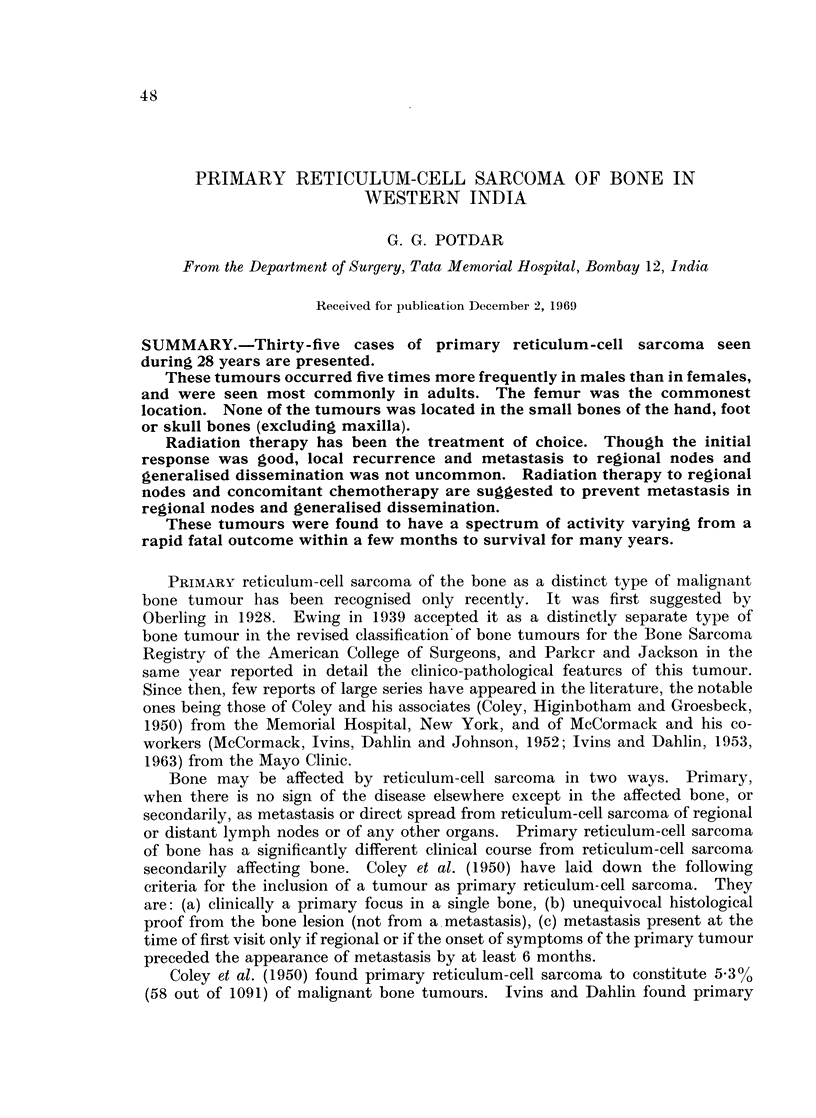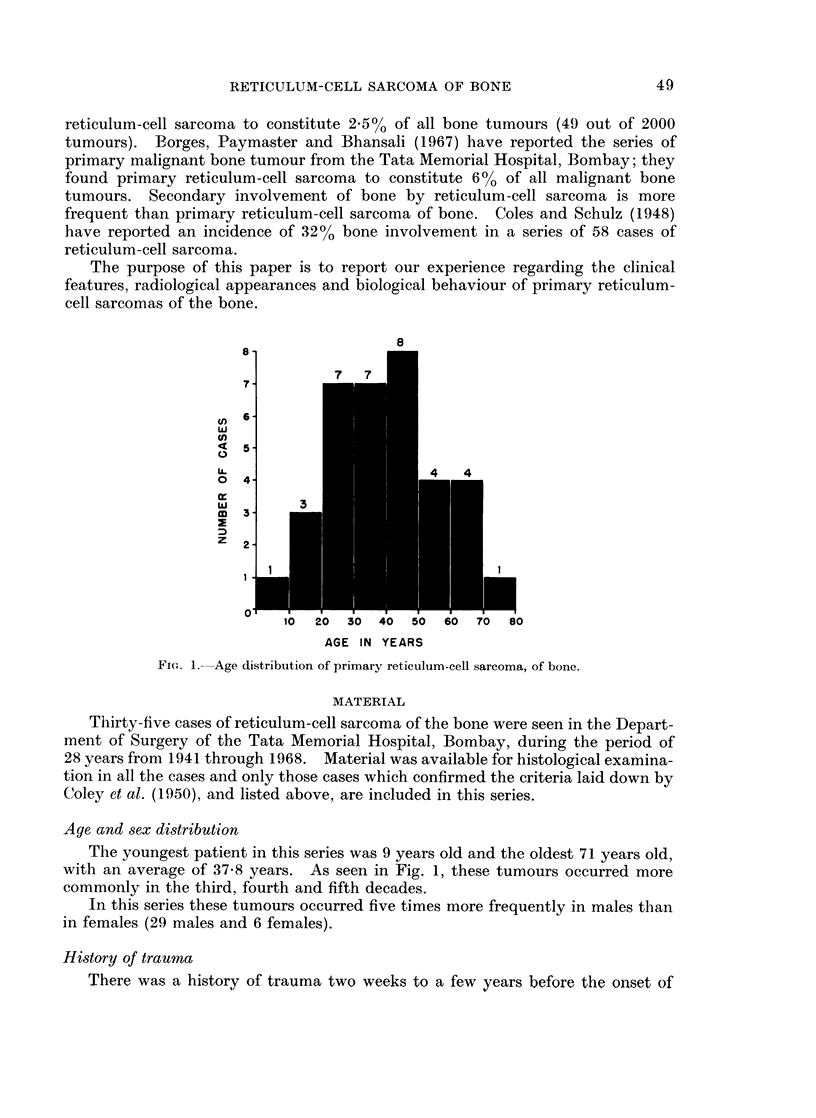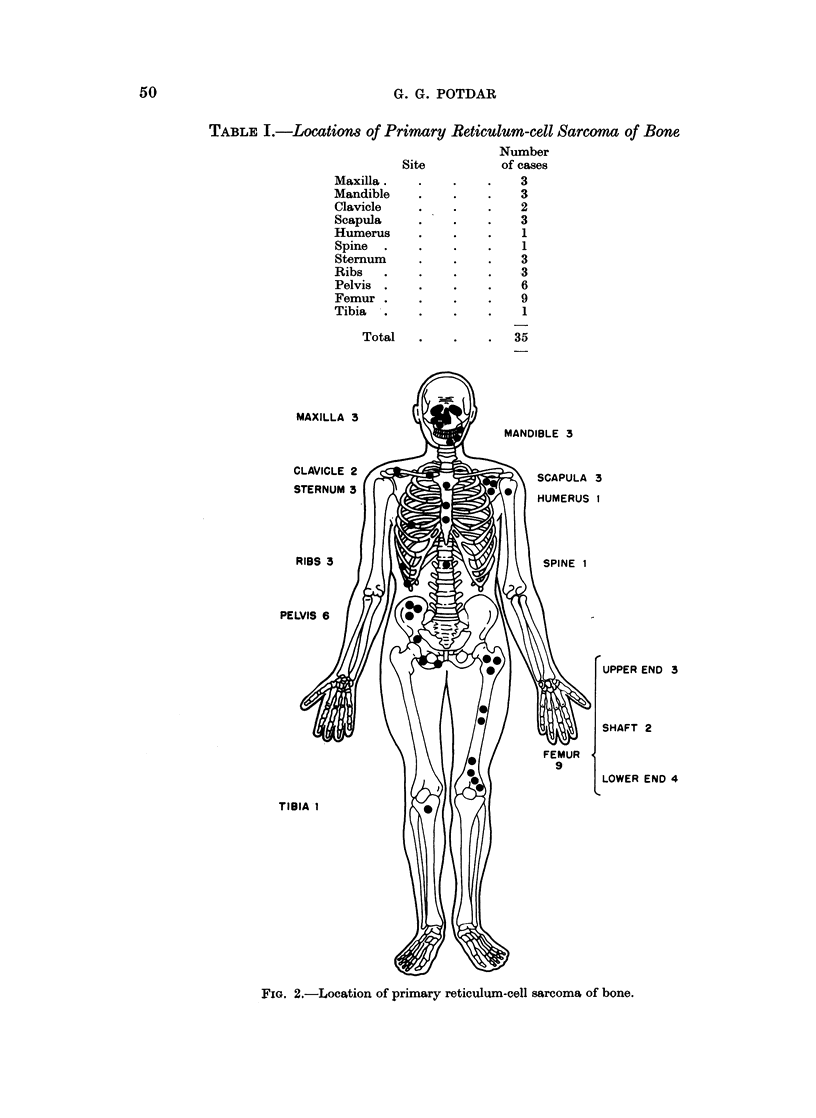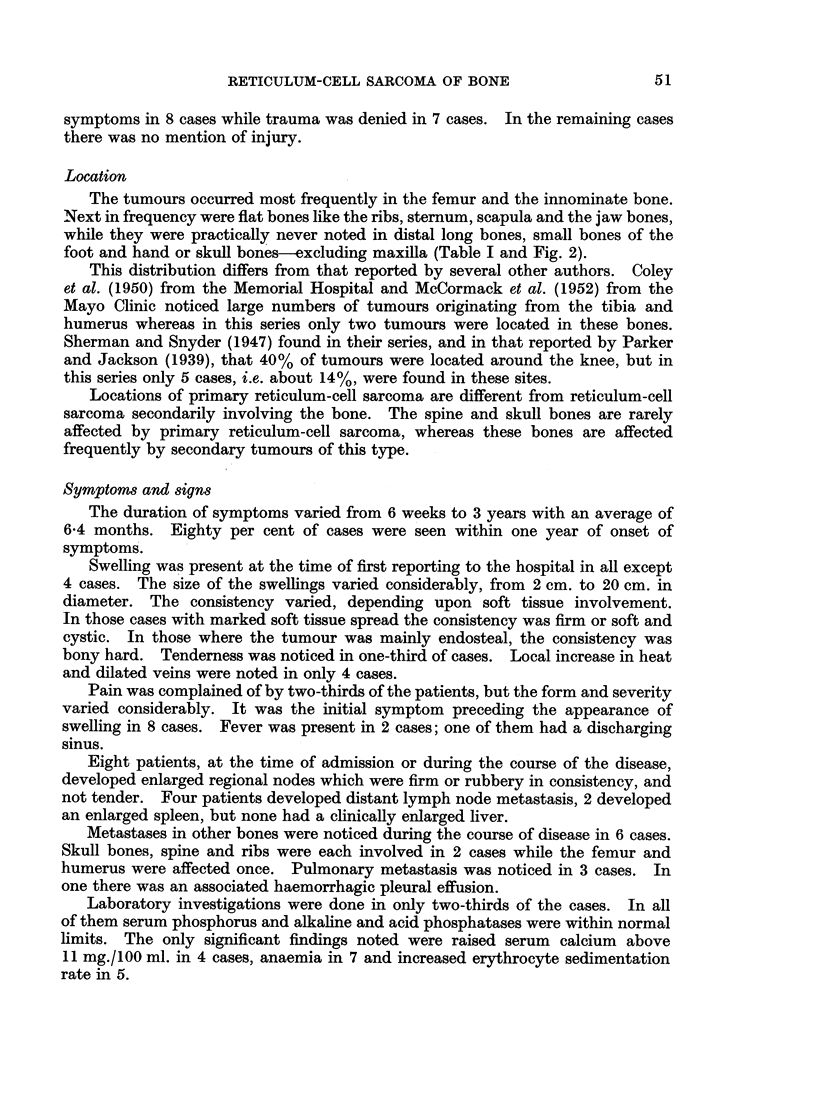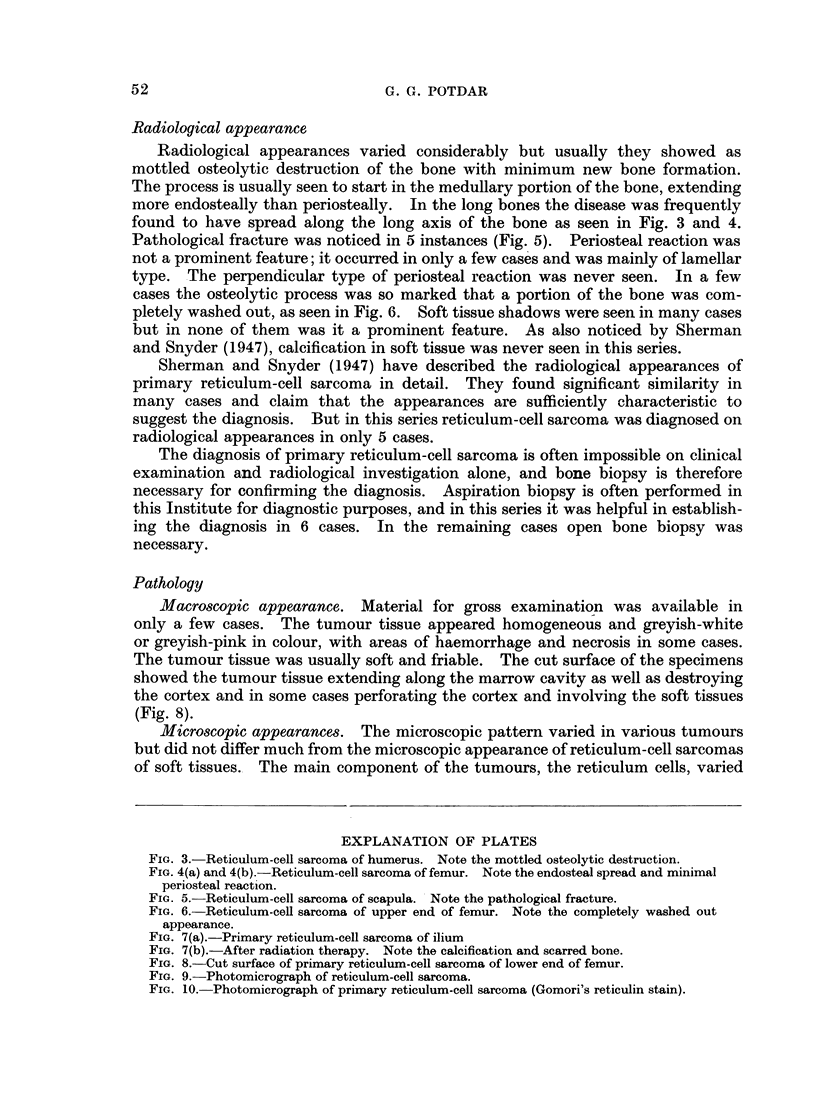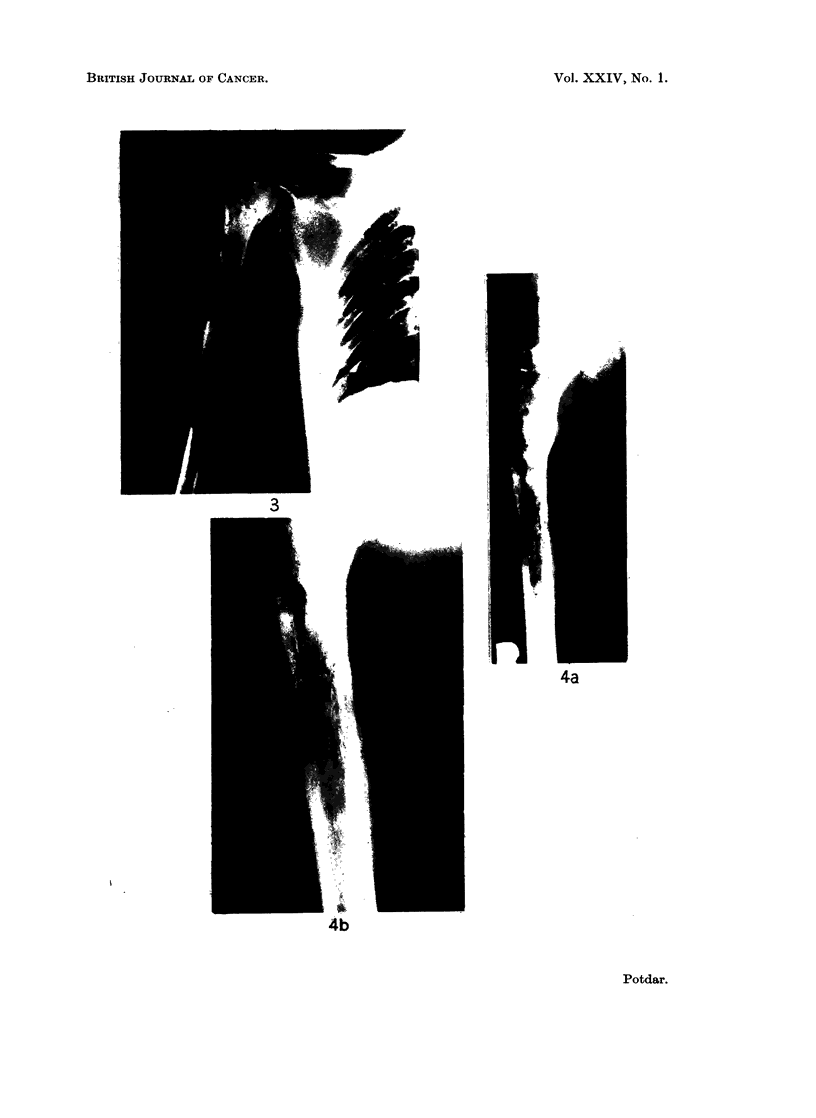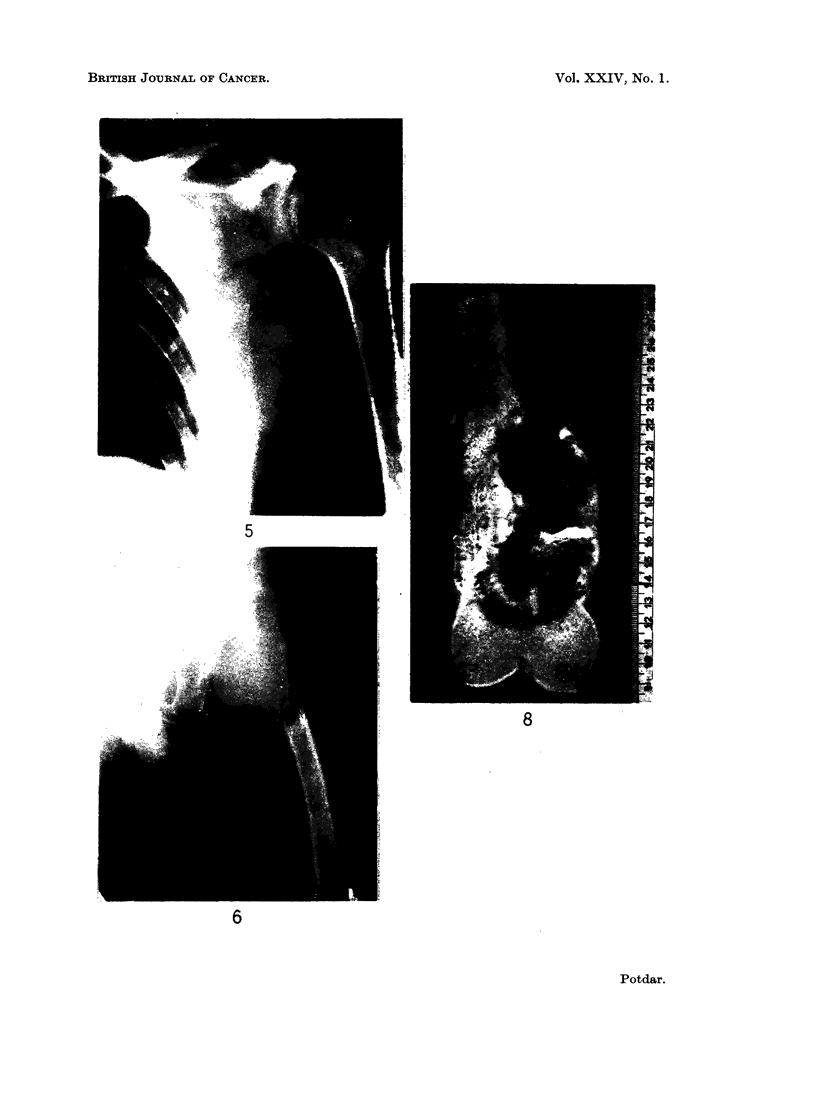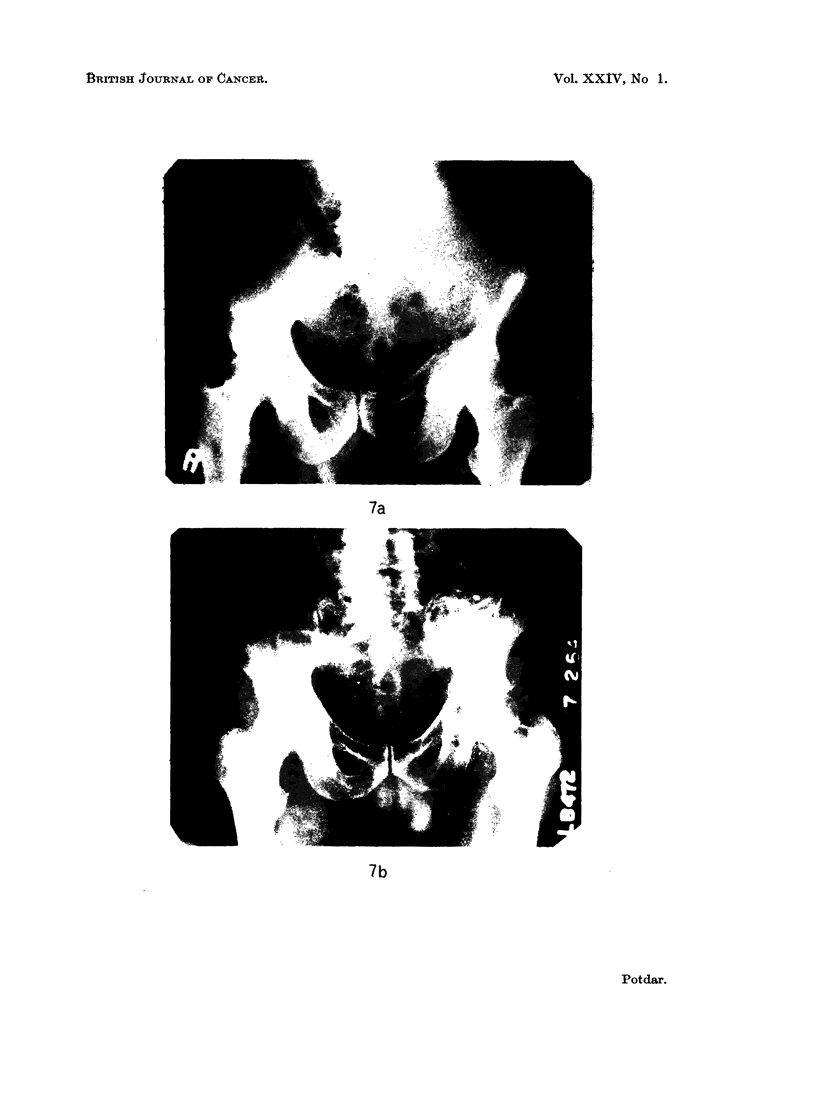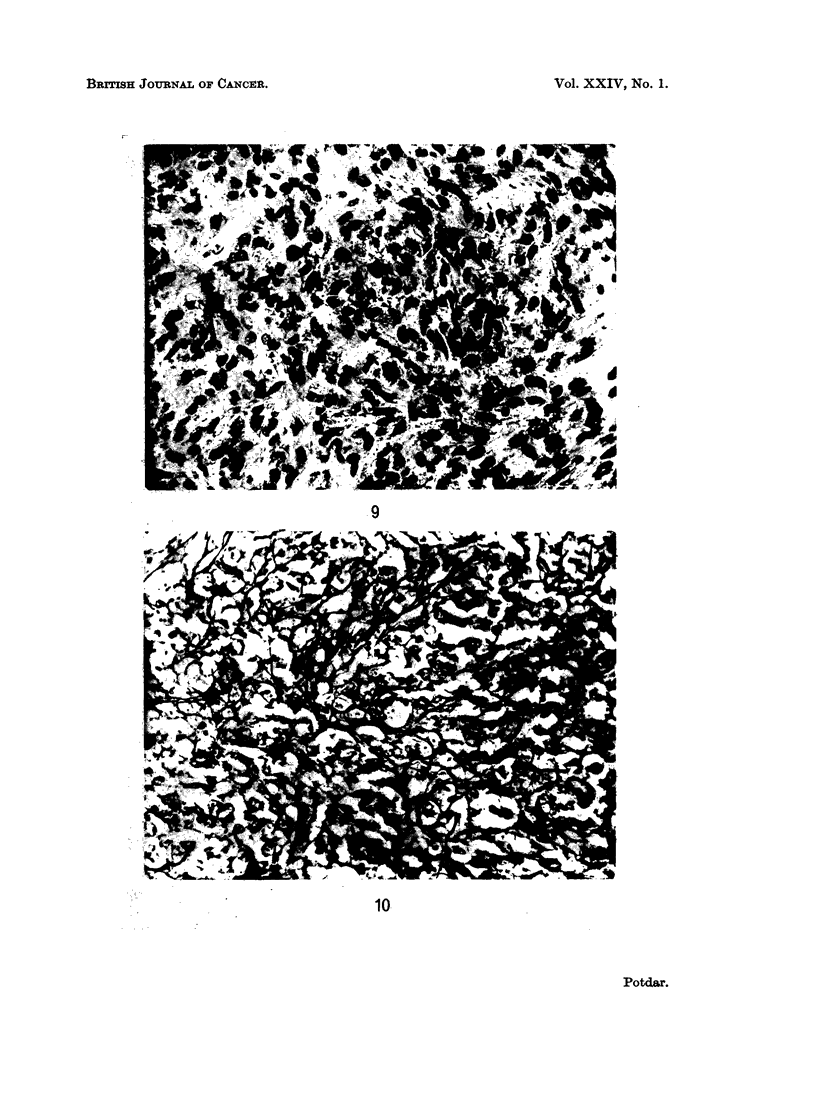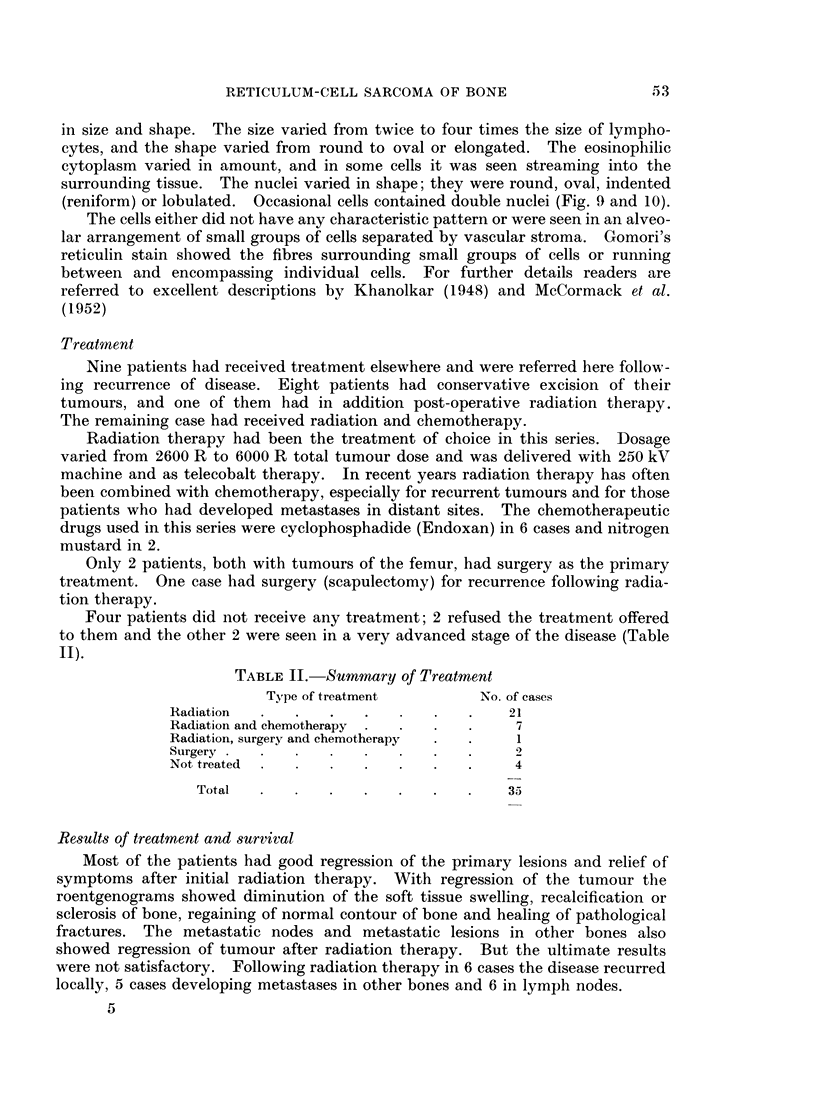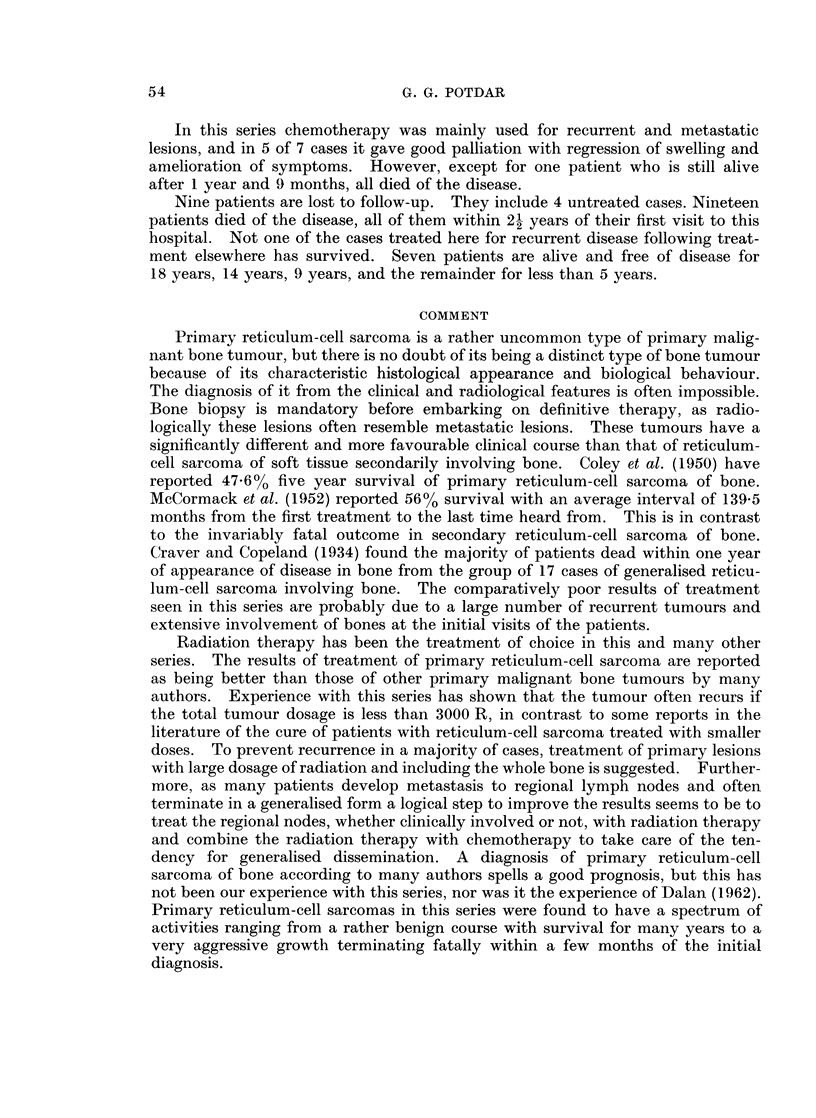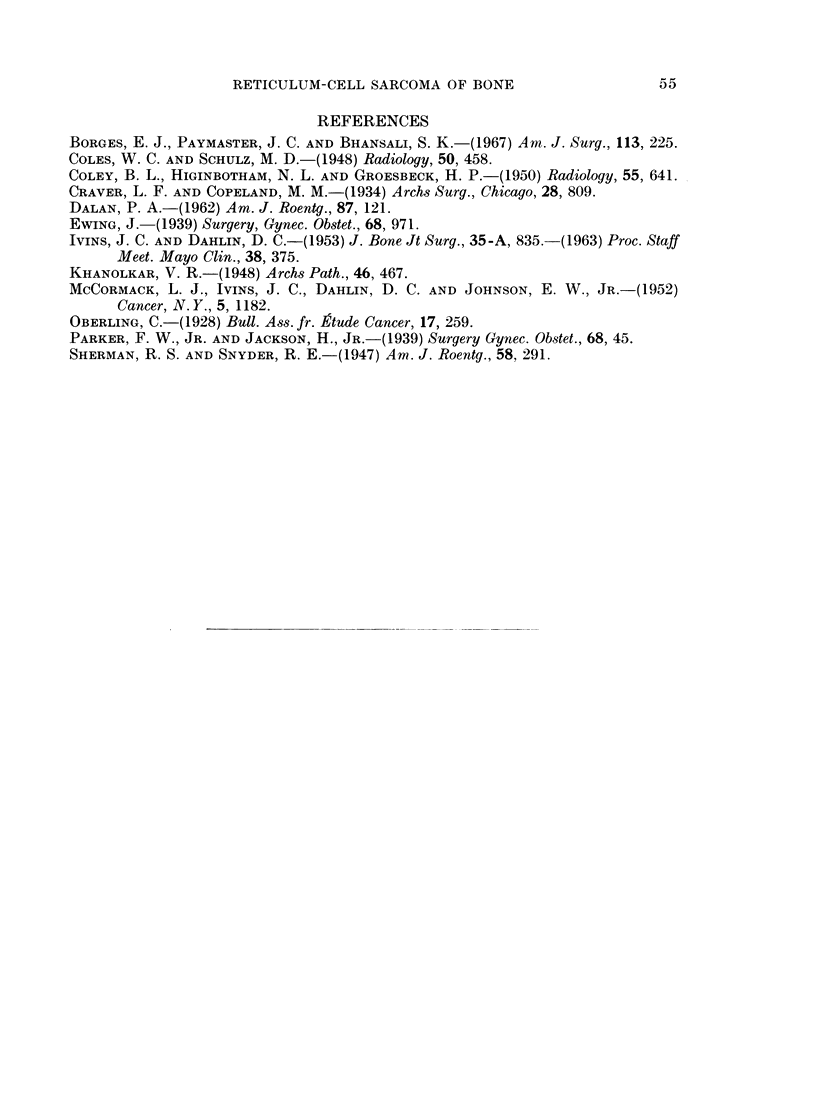# Primary Reticulum-Cell Sarcoma of Bone in Western India

**DOI:** 10.1038/bjc.1970.7

**Published:** 1970-03

**Authors:** G. G. Potdar

## Abstract

**Images:**


### PRIMARY RETICULUM-CELL SARCOMA OF BONE IN WESTERN INDIA

G. G. POTDAR

*From the Department of Surgery, Tata Memorial Hospital, Bombay 12, India*

Received for publication December 2, 1969

**SUMMARY.—Thirty-five cases of primary reticulum-cell sarcoma seen during 28 years are presented.**

**These tumours occurred five times more frequently in males than in females, and were seen most commonly in adults. The femur was the commonest location. None of the tumours was located in the small bones of the hand, foot or skull bones (excluding maxilla).**

**Radiation therapy has been the treatment of choice. Though the initial response was good, local recurrence and metastasis to regional nodes and generalised dissemination was not uncommon. Radiation therapy to regional nodes and concomitant chemotherapy are suggested to prevent metastasis in regional nodes and generalised dissemination.**

**These tumours were found to have a spectrum of activity varying from a rapid fatal outcome within a few months to survival for many years.**

PRIMARY reticulum-cell sarcoma of the bone as a distinct type of malignant bone tumour has been recognised only recently. It was first suggested by Oberling in 1928. Ewing in 1939 accepted it as a distinctly separate type of bone tumour in the revised classification of bone tumours for the Bone Sarcoma Registry of the American College of Surgeons, and Parker and Jackson in the same year reported in detail the clinico-pathological features of this tumour. Since then, few reports of large series have appeared in the literature, the notable ones being those of Coley and his associates (Coley, Higinbotham and Groesbeck, 1950) from the Memorial Hospital, New York, and of McCormack and his co-workers (McCormack, Ivins, Dahlin and Johnson, 1952; Ivins and Dahlin, 1953, 1963) from the Mayo Clinic.

Bone may be affected by reticulum-cell sarcoma in two ways. Primary, when there is no sign of the disease elsewhere except in the affected bone, or secondarily, as metastasis or direct spread from reticulum-cell sarcoma of regional or distant lymph nodes or of any other organs. Primary reticulum-cell sarcoma of bone has a significantly different clinical course from reticulum-cell sarcoma secondarily affecting bone. Coley *et al.* (1950) have laid down the following criteria for the inclusion of a tumour as primary reticulum-cell sarcoma. They are: (a) clinically a primary focus in a single bone, (b) unequivocal histological proof from the bone lesion (not from a metastasis), (c) metastasis present at the time of first visit only if regional or if the onset of symptoms of the primary tumour preceded the appearance of metastasis by at least 6 months.

Coley *et al.* (1950) found primary reticulum-cell sarcoma to constitute 5·3% (58 out of 1091) of malignant bone tumours. Ivins and Dahlin found primary reticulum-cell sarcoma to constitute 2·5% of all bone tumours (49 out of 2000 tumours). Borges, Paymaster and Bhansali (1967) have reported the series of primary malignant bone tumour from the Tata Memorial Hospital, Bombay; they found primary reticulum-cell sarcoma to constitute 6% of all malignant bone tumours. Secondary involvement of bone by reticulum-cell sarcoma is more frequent than primary reticulum-cell sarcoma of bone. Coles and Schulz (1948) have reported an incidence of 32% bone involvement in a series of 58 cases of reticulum-cell sarcoma.

The purpose of this paper is to report our experience regarding the clinical features, radiological appearances and biological behaviour of primary reticulum-cell sarcomas of the bone.

FIG. 1.—Age distribution of primary reticulum-cell sarcoma, of bone.

### MATERIAL

Thirty-five cases of reticulum-cell sarcoma of the bone were seen in the Department of Surgery of the Tata Memorial Hospital, Bombay, during the period of 28 years from 1941 through 1968. Material was available for histological examination in all the cases and only those cases which confirmed the criteria laid down by Coley *et al.* (1950), and listed above, are included in this series.

### *Age and sex distribution*

The youngest patient in this series was 9 years old and the oldest 71 years old, with an average of 37·8 years. As seen in Fig. 1, these tumours occurred more commonly in the third, fourth and fifth decades.

In this series these tumours occurred five times more frequently in males than in females (29 males and 6 females).

### *History of trauma*

There was a history of trauma two weeks to a few years before the onset of

TABLE I.—*Locations of Primary Reticulum-cell Sarcoma of Bone*

| Site | Number of cases |
|---|---|
| Maxilla | 3 |
| Mandible | 3 |
| Clavicle | 2 |
| Scapula | 3 |
| Humerus | 1 |
| Spine | 1 |
| Sternum | 3 |
| Ribs | 3 |
| Pelvis | 6 |
| Femur | 9 |
| Tibia | 1 |
| Total | 35 |

FIG. 2.—Location of primary reticulum-cell sarcoma of bone.

symptoms in 8 cases while trauma was denied in 7 cases. In the remaining cases there was no mention of injury.

*Location*

The tumours occurred most frequently in the femur and the innominate bone. Next in frequency were flat bones like the ribs, sternum, scapula and the jaw bones, while they were practically never noted in distal long bones, small bones of the foot and hand or skull bones—excluding maxilla (Table I and Fig. 2).

This distribution differs from that reported by several other authors. Coley *et al.* (1950) from the Memorial Hospital and McCormack *et al.* (1952) from the Mayo Clinic noticed large numbers of tumours originating from the tibia and humerus whereas in this series only two tumours were located in these bones. Sherman and Snyder (1947) found in their series, and in that reported by Parker and Jackson (1939), that 40% of tumours were located around the knee, but in this series only 5 cases, *i.e.* about 14%, were found in these sites.

Locations of primary reticulum-cell sarcoma are different from reticulum-cell sarcoma secondarily involving the bone. The spine and skull bones are rarely affected by primary reticulum-cell sarcoma, whereas these bones are affected frequently by secondary tumours of this type.

*Symptoms and signs*

The duration of symptoms varied from 6 weeks to 3 years with an average of 6·4 months. Eighty per cent of cases were seen within one year of onset of symptoms.

Swelling was present at the time of first reporting to the hospital in all except 4 cases. The size of the swellings varied considerably, from 2 cm. to 20 cm. in diameter. The consistency varied, depending upon soft tissue involvement. In those cases with marked soft tissue spread the consistency was firm or soft and cystic. In those where the tumour was mainly endosteal, the consistency was bony hard. Tenderness was noticed in one-third of cases. Local increase in heat and dilated veins were noted in only 4 cases.

Pain was complained of by two-thirds of the patients, but the form and severity varied considerably. It was the initial symptom preceding the appearance of swelling in 8 cases. Fever was present in 2 cases; one of them had a discharging sinus.

Eight patients, at the time of admission or during the course of the disease, developed enlarged regional nodes which were firm or rubbery in consistency, and not tender. Four patients developed distant lymph node metastasis, 2 developed an enlarged spleen, but none had a clinically enlarged liver.

Metastases in other bones were noticed during the course of disease in 6 cases. Skull bones, spine and ribs were each involved in 2 cases while the femur and humerus were affected once. Pulmonary metastasis was noticed in 3 cases. In one there was an associated haemorrhagic pleural effusion.

Laboratory investigations were done in only two-thirds of the cases. In all of them serum phosphorus and alkaline and acid phosphatases were within normal limits. The only significant findings noted were raised serum calcium above 11 mg./100 ml. in 4 cases, anaemia in 7 and increased erythrocyte sedimentation rate in 5.

*Radiological appearance*

Radiological appearances varied considerably but usually they showed as mottled osteolytic destruction of the bone with minimum new bone formation. The process is usually seen to start in the medullary portion of the bone, extending more endosteally than periosteally. In the long bones the disease was frequently found to have spread along the long axis of the bone as seen in Fig. 3 and 4. Pathological fracture was noticed in 5 instances (Fig. 5). Periosteal reaction was not a prominent feature; it occurred in only a few cases and was mainly of lamellar type. The perpendicular type of periosteal reaction was never seen. In a few cases the osteolytic process was so marked that a portion of the bone was completely washed out, as seen in Fig. 6. Soft tissue shadows were seen in many cases but in none of them was it a prominent feature. As also noticed by Sherman and Snyder (1947), calcification in soft tissue was never seen in this series.

Sherman and Snyder (1947) have described the radiological appearances of primary reticulum-cell sarcoma in detail. They found significant similarity in many cases and claim that the appearances are sufficiently characteristic to suggest the diagnosis. But in this series reticulum-cell sarcoma was diagnosed on radiological appearances in only 5 cases.

The diagnosis of primary reticulum-cell sarcoma is often impossible on clinical examination and radiological investigation alone, and bone biopsy is therefore necessary for confirming the diagnosis. Aspiration biopsy is often performed in this Institute for diagnostic purposes, and in this series it was helpful in establishing the diagnosis in 6 cases. In the remaining cases open bone biopsy was necessary.

*Pathology*

*Macroscopic appearance.* Material for gross examination was available in only a few cases. The tumour tissue appeared homogeneous and greyish-white or greyish-pink in colour, with areas of haemorrhage and necrosis in some cases. The tumour tissue was usually soft and friable. The cut surface of the specimens showed the tumour tissue extending along the marrow cavity as well as destroying the cortex and in some cases perforating the cortex and involving the soft tissues (Fig. 8).

*Microscopic appearances.* The microscopic pattern varied in various tumours but did not differ much from the microscopic appearance of reticulum-cell sarcomas of soft tissues. The main component of the tumours, the reticulum cells, varied

---

EXPLANATION OF PLATES

FIG. 3.—Reticulum-cell sarcoma of humerus. Note the mottled osteolytic destruction.

FIG. 4(a) and 4(b).—Reticulum-cell sarcoma of femur. Note the endosteal spread and minimal periosteal reaction.

FIG. 5.—Reticulum-cell sarcoma of scapula. Note the pathological fracture.

FIG. 6.—Reticulum-cell sarcoma of upper end of femur. Note the completely washed out appearance.

FIG. 7(a).—Primary reticulum-cell sarcoma of ilium

FIG. 7(b).—After radiation therapy. Note the calcification and scarred bone.

FIG. 8.—Cut surface of primary reticulum-cell sarcoma of lower end of femur.

FIG. 9.—Photomicrograph of reticulum-cell sarcoma.

FIG. 10.—Photomicrograph of primary reticulum-cell sarcoma (Gomori's reticulin stain).

3

4a

4b

5

6

8

7a

7b

9

10

in size and shape. The size varied from twice to four times the size of lymphocytes, and the shape varied from round to oval or elongated. The eosinophilic cytoplasm varied in amount, and in some cells it was seen streaming into the surrounding tissue. The nuclei varied in shape; they were round, oval, indented (reniform) or lobulated. Occasional cells contained double nuclei (Fig. 9 and 10).

The cells either did not have any characteristic pattern or were seen in an alveolar arrangement of small groups of cells separated by vascular stroma. Gomori's reticulin stain showed the fibres surrounding small groups of cells or running between and encompassing individual cells. For further details readers are referred to excellent descriptions by Khanolkar (1948) and McCormack *et al.* (1952)

*Treatment*

Nine patients had received treatment elsewhere and were referred here following recurrence of disease. Eight patients had conservative excision of their tumours, and one of them had in addition post-operative radiation therapy. The remaining case had received radiation and chemotherapy.

Radiation therapy had been the treatment of choice in this series. Dosage varied from 2600 R to 6000 R total tumour dose and was delivered with 250 kV machine and as telecobalt therapy. In recent years radiation therapy has often been combined with chemotherapy, especially for recurrent tumours and for those patients who had developed metastases in distant sites. The chemotherapeutic drugs used in this series were cyclophosphadide (Endoxan) in 6 cases and nitrogen mustard in 2.

Only 2 patients, both with tumours of the femur, had surgery as the primary treatment. One case had surgery (scapulectomy) for recurrence following radiation therapy.

Four patients did not receive any treatment; 2 refused the treatment offered to them and the other 2 were seen in a very advanced stage of the disease (Table II).

TABLE II.—*Summary of Treatment*

| Type of treatment | No. of cases |
|---|---|
| Radiation . . . . . . . . | 21 |
| Radiation and chemotherapy . . . . | 7 |
| Radiation, surgery and chemotherapy . . | 1 |
| Surgery . . . . . . . . . | 2 |
| Not treated . . . . . . . . | 4 |
| Total . . . . . . . . | 35 |

*Results of treatment and survival*

Most of the patients had good regression of the primary lesions and relief of symptoms after initial radiation therapy. With regression of the tumour the roentgenograms showed diminution of the soft tissue swelling, recalcification or sclerosis of bone, regaining of normal contour of bone and healing of pathological fractures. The metastatic nodes and metastatic lesions in other bones also showed regression of tumour after radiation therapy. But the ultimate results were not satisfactory. Following radiation therapy in 6 cases the disease recurred locally, 5 cases developing metastases in other bones and 6 in lymph nodes.

In this series chemotherapy was mainly used for recurrent and metastatic lesions, and in 5 of 7 cases it gave good palliation with regression of swelling and amelioration of symptoms. However, except for one patient who is still alive after 1 year and 9 months, all died of the disease.

Nine patients are lost to follow-up. They include 4 untreated cases. Nineteen patients died of the disease, all of them within $2\frac{1}{2}$ years of their first visit to this hospital. Not one of the cases treated here for recurrent disease following treatment elsewhere has survived. Seven patients are alive and free of disease for 18 years, 14 years, 9 years, and the remainder for less than 5 years.

### COMMENT

Primary reticulum-cell sarcoma is a rather uncommon type of primary malignant bone tumour, but there is no doubt of its being a distinct type of bone tumour because of its characteristic histological appearance and biological behaviour. The diagnosis of it from the clinical and radiological features is often impossible. Bone biopsy is mandatory before embarking on definitive therapy, as radiologically these lesions often resemble metastatic lesions. These tumours have a significantly different and more favourable clinical course than that of reticulum-cell sarcoma of soft tissue secondarily involving bone. Coley *et al.* (1950) have reported 47·6% five year survival of primary reticulum-cell sarcoma of bone. McCormack *et al.* (1952) reported 56% survival with an average interval of 139·5 months from the first treatment to the last time heard from. This is in contrast to the invariably fatal outcome in secondary reticulum-cell sarcoma of bone. Craver and Copeland (1934) found the majority of patients dead within one year of appearance of disease in bone from the group of 17 cases of generalised reticulum-cell sarcoma involving bone. The comparatively poor results of treatment seen in this series are probably due to a large number of recurrent tumours and extensive involvement of bones at the initial visits of the patients.

Radiation therapy has been the treatment of choice in this and many other series. The results of treatment of primary reticulum-cell sarcoma are reported as being better than those of other primary malignant bone tumours by many authors. Experience with this series has shown that the tumour often recurs if the total tumour dosage is less than 3000 R, in contrast to some reports in the literature of the cure of patients with reticulum-cell sarcoma treated with smaller doses. To prevent recurrence in a majority of cases, treatment of primary lesions with large dosage of radiation and including the whole bone is suggested. Furthermore, as many patients develop metastasis to regional lymph nodes and often terminate in a generalised form a logical step to improve the results seems to be to treat the regional nodes, whether clinically involved or not, with radiation therapy and combine the radiation therapy with chemotherapy to take care of the tendency for generalised dissemination. A diagnosis of primary reticulum-cell sarcoma of bone according to many authors spells a good prognosis, but this has not been our experience with this series, nor was it the experience of Dalan (1962). Primary reticulum-cell sarcomas in this series were found to have a spectrum of activities ranging from a rather benign course with survival for many years to a very aggressive growth terminating fatally within a few months of the initial diagnosis.

### REFERENCES

Borges, E. J., Paymaster, J. C. and Bhansali, S. K.—(1967) *Am. J. Surg.*, **113**, 225.
Coles, W. C. and Schulz, M. D.—(1948) *Radiology*, **50**, 458.
Coley, B. L., Higinbotham, N. L. and Groesbeck, H. P.—(1950) *Radiology*, **55**, 641.
Craver, L. F. and Copeland, M. M.—(1934) *Archs Surg., Chicago*, **28**, 809.
Dalan, P. A.—(1962) *Am. J. Roentg.*, **87**, 121.
Ewing, J.—(1939) *Surgery, Gynec. Obstet.*, **68**, 971.
Ivins, J. C. and Dahlin, D. C.—(1953) *J. Bone Jt Surg.*, **35-A**, 835.—(1963) *Proc. Staff Meet. Mayo Clin.*, **38**, 375.
Khanolkar, V. R.—(1948) *Archs Path.*, **46**, 467.
McCormack, L. J., Ivins, J. C., Dahlin, D. C. and Johnson, E. W., Jr.—(1952) *Cancer, N.Y.*, **5**, 1182.
Oberling, C.—(1928) *Bull. Ass. fr. Étude Cancer*, **17**, 259.
Parker, F. W., Jr. and Jackson, H., Jr.—(1939) *Surgery Gynec. Obstet.*, **68**, 45.
Sherman, R. S. and Snyder, R. E.—(1947) *Am. J. Roentg.*, **58**, 291.